# Relationships between inappropriate eating habits and problematic alcohol use, cigarette and waterpipe dependence among male adolescents in Lebanon

**DOI:** 10.1186/s12889-021-10184-2

**Published:** 2021-01-15

**Authors:** Jana Berro, Marwan Akel, Souheil Hallit, Sahar Obeid

**Affiliations:** 1 Faculty of Arts & Sciences, Lebanese American University, Byblos, Lebanon; 2 School of Pharmacy, Lebanese International University, Beirut, Lebanon; 3 INSPECT-LB: Institut National de Santé Publique, Epidemiologie Clinique et Toxicologie-Liban, Beirut, Lebanon; 4 Faculty of Medicine and Medical Sciences, Holy Spirit University of Kaslik (USEK), Jounieh, Lebanon; 5 Faculty of Arts and Sciences, Holy Spirit University of Kaslik (USEK), Jounieh, Lebanon

**Keywords:** Eating attitudes, Alcohol, Cigarette dependence, Waterpipe dependence, Lebanon, Adolescents, Male gender

## Abstract

**Background:**

it is important to investigate the relationship between disordered eating in male adolescents and smoking and alcohol consumption as they are risk factors to other diseases as well. For example, high levels of alcohol accompanied by the acidic damage and nutritional deficit exhibited in people with disordered eating habits - due to induced vomiting - has been shown to increase risk of esophageal cancer. Considering the very few studies done on disordered eating prevailing in males and the prevalence of smoking and drinking habits, our study aims to investigate the correlation between inappropriate eating habits and one’s dependence on cigarettes, waterpipes, and alcohol all the while focusing on male adolescents.

**Methods:**

This was a cross-sectional observational study that enrolled 389 male students (13–17 years of age) drawn from five Lebanese schools between October and December 2019.

**Results:**

The MANCOVA analysis was performed taking the addiction scales as the dependent variables and the EAT-26 score as an independent variable, adjusting for the covariates (age, BMI and household crowding index). Higher EAT-26 scores (more inappropriate eating attitudes) were significantly associated with higher waterpipe dependence (B = 0.11; CI 0.06–0.17) and more problematic alcohol use (B = 0.08; CI 0.04–0.12), but not cigarette dependence.

**Conclusions:**

This study revealed an association between inappropriate eating and increased drinking and smoking the waterpipe, but not cigarettes. The manifestation of inappropriate eating habits was found to be significant among male adolescents; in the literature, this is also true for female adolescents. In Lebanon, the engagement of adolescents in alcohol drinking and waterpipe smoking is frequent as they are accessible due to the lack of law enforcement and supervision in the country. In addition, while this study found an association between inappropriate eating habits and dependency on smoking and drinking, further investigation should be done on the role of one’s psychology in their exhibition of disordered eating as means to prevent the manifestation of these habits.

## Background

Eating habits vary greatly from one person to another where inappropriate habits range from dieting to overeating [1]. Generally, males have poorer and less healthy lifestyles than females [2]. However, disordered eating habits, usually in attempt of weight control, are more common among female than among male adolescents, with the prevalence being 50.7 and 33.7%, respectively [3]. Healthy weight is misconstrued among high school students thus, disordered eating behaviors are prominent among adolescents [4, 5]. Not only have disordered eating patterns been linked to psychological issues [6], Patton et al. suggested that dieting, for example, increases the risk of developing eating disorders in later stages of life [7]. Furthermore, eating disorders include a spectrum of numerous disorders, the most common ones being anorexia nervosa and bulimia nervosa [8]. Anorexia is a condition where people starve themselves whereas bulimia is a condition where they alternate between starving them and bingeing, proceeded by purging, i.e. removing calories from the body [9]. Each eating disorder varies in terms of their prevalence in one gender. For example, anorexia is usually prevalent in females rather than males partly due to the pressures imposed by society’s standards in regards to women looking ‘thin’ and ‘slim’ [10]. In contrast, women seem less likely to experience subthreshold binge eating disorder than men [10, 11]; overweight and obesity are, hence, more common among males [12].

Even though disordered eating habits have a huge impact on one’s psychology, it is important to investigate its effect on one’s dependency on smoking – cigarettes and water pipe – and alcohol in Lebanon. Not only do those with higher Body Mass Index (BMI) have a higher chance of consuming tobacco and smoking [13, 14], but also, non-smokers have healthier eating behaviors than smokers. Furthermore, relationships were established between an increase in the amount of alcohol consumed and an increase in BMI [15]. However, those who drank frequently but in smaller amounts had a lower BMI [16]. In fact, the link between alcoholism and eating disorders, such as anorexia and bulimia nervosa, was found to be bidirectional [17].

Most studies focus on the effect of disordered eating on females as they experience these behaviors more frequently than males. Disordered eating attitudes studied among females were associated with an increase in cigarette smoking [18]. For example, those who practiced purging or dieting were more likely to drink alcohol and suffer the negative consequences of heavy drinking than women who did not engage in these eating behaviors [19]. Additionally, women who suffered from disordered eating were at higher risk of involving themselves in heavy drinking [20]. It was suggested that one of the main reasons behind the association of binge eating and alcohol drinking is that both behaviors serve as a coping mechanism, mainly avoidance [21]. The focus of most studies on the aforementioned topic could be due to the difference between females and males in terms of compensatory behavior, thus leading to disordered eating practices to be underestimated among males.

Hence, it is important to further investigate this topic among males. Furthermore, it has been suggested that the eating behaviors of non-smoking males are as healthy as those of female smokers [13]. In addition, males are more likely to smoke and drink than females. In Lebanon, waterpipe use was more prominent among males than females [22]. The difference in gender habits could be due to the higher social acceptability of smoking in males compared to females, particularly in the patriarchal society of Lebanon. Conservative views might be held more strongly towards cigarettes than water pipe [23, 24]. Furthermore, males were found to experience a higher consumption of alcohol than females in addition to being at higher risk of developing alcohol use disorder [25, 26].

Not only is it a necessity to focus our study on males, but also on male adolescents in high school across Lebanon. Firstly, the engagement of people their age in disordered eating behaviors put them at higher risk of developing eating disorders and other health-risk behaviors [27]. Smoking both cigarettes and water pipe in Lebanon among adolescents is on the rise. Around 35% of 13 to 15-year olds have already tried the water pipe [28] with 19% of adolescents regularly using the waterpipe. Lebanon ranks the highest in terms of smoking frequency and intensity in the Middle East [29]. Furthermore, adolescents tend to perceive water pipes as more appealing due to its different flavor options and their misconception of it being less harmful than cigarettes [30].

Alcohol is another phenomenon posing a health concern worldwide as it is increasing among adolescents [31]. Furthermore, in Lebanon, alcohol is more accessible to adolescents in comparison to other countries, due to its cheap price and the lack of law enforcement that prevents adolescents from purchasing alcohol [32]. Lebanon has witnessed an increase of 48% between 2005 and 2011 in adolescents’ drunkenness [33]; this might be due to several reasons including advertisement on social media combined with their feeling of power or ability to cope with stress in the presence of alcohol [34, 35].

Furthermore, it is important to investigate the relationship between disordered eating and smoking and alcohol use within adolescents because alcohol and smoking and eating disorders are risk factors to other diseases as well. For example, high levels of alcohol accompanied by the acidic damage and nutritional deficit exhibited in people with disordered eating habits - due to induced vomiting - has been shown to increase risk of esophageal cancer [36]. Smoking has also been established to be a risk factor for esophageal cancer [37] and osteoporosis, which is often occurring with people with Anorexia Nervosa [38].

While multiple studies have been done investigating how different factors – such us low earnings, weaker labor market attachments, genetic factors [39], attachment styles [40] and parental separation [41] – are associated with smoking and alcohol consumption, few studies have been done to investigate the relationship between disordered eating and the prevalence of smoking and drinking among males. While the relationship might be bidirectional [17, 42], our study aims to investigate how inappropriate eating habits play a role in one’s dependence on cigarettes, waterpipes, and alcohol in male adolescents. In addition, the establishment of this relationship has clinical implication for the selection of treatment methods for eating disorders; practitioners should know whether to focus on one’s dependence.

## Methods

### Study design and procedure

This was a cross-sectional observational study that enrolled 389 male students drawn from five Lebanese schools between October and December 2019. A list of the schools available in each Lebanese district was provided by the Ministry of Education and Higher Education; one school was chosen from each district using a simple randomization technique; the districts included the capital Beirut, Mount Lebanon, North, Beqaa, and South. A list of students was obtained from the designated school; all male students from grades 9, 10, 11 and 12 (13–17 years of age) at each school were asked to participate (total *N* = 500). Students were allowed to fill the questionnaires on a voluntary basis and the survey was administered in classrooms to avoid parents’ influence. Subjects who refused to complete the questionnaire were excluded. Any personal identification was removed from the questionnaire before coding began.

### Minimal sample size calculation

According to the G-power software, and based on an effect size f2 = 4%, an alpha error of 5%, a power of 80%, and taking into consideration 4 factors to be entered in the multivariable analysis, the results showed that a minimal number of 304 was needed.

### Data collection and measures

The data collection sheet used to retrieve data from the participants was established based on validated and standardized questionnaires [22, 25–28]. The survey questionnaire was self-report, in Arabic, and distributed as a paper copy. Before use, the questionnaire was translated into Arabic (process involving two independent translations, synthesis of the two translations, back translations, review of the pre-final version and pretesting). The questionnaire included two sections. The first section collected demographic information, including participant’s age and socioeconomic characteristics. The second part was dedicated for the assessment of eating attitude and addiction (alcohol, nicotine and waterpipe addiction).

**Eating Attitude Test (EAT-26):** The EAT, validated in Lebanon [43], was used for the assessment of disordered/inappropriate eating attitudes [44]. It includes 26 questions scored from infrequently/almost never/never (0) to always (3). Scores ≥20 reflect probable disordered/inappropriate eating attitudes [45] (α Cronbach in this study = 0.910).

For the assessment of alcohol addiction, the validated **Alcohol Use Disorders Identification Test** (AUDIT) scale was used. This tool is composed of 10 items to assess alcohol use, drinking patterns, and alcohol-related issues, which can be administered by a clinician or self-administered [46]. Scores ≥8 reflect high risk of alcohol use disorder (α Cronbach = 0.861). This scale was recently validated in Lebanon [47].

The **Fagerström Test for Nicotine Dependence** (FTND) is a six-item instrument used to screen for addiction to cigarette smoking. Items are scored as 0/1 for questions with a yes/no answer and 0–3 for multiple-choice items. Higher scores reflect more nicotine dependence [48] (α Cronbach in this study = 0.874).

The **Lebanese Waterpipe Dependence Scale-11** (LWDS-11) test was used to assess waterpipe dependence [49]. The LWDS-11 is composed of 11 items, measured on a 4-point Likert scale ranging from 0 to 3. The total scale is calculated by summing the 11 items. In this study, the Cronbach’s alpha was 0.908.

### Statistical analysis

SPSS software version 25 was used to conduct data analysis. Since the LWDS, FTND and AUDIT scores showed a non-normal distribution, Spearman correlation was used to evaluate the association between continuous variables. A multivariate analysis of covariance (MANCOVA) was conducted taking the LWDS-11, FTND and AUDIT scores as a dependent variables and the EAT-26 score taken as the independent variable, after adjusting over other confounding variables: age, BMI and household crowding index. A partial eta squared of │0.01–0.05│indicated a small effect, │0.06–0.13│a moderate effect and > │0.14│ a large effect. A *p* < 0.05 was considered significant.

## Results

### Sociodemographic and other characteristics of the adolescents

A total of 389 (77.8%) out of 500 students accepted to participate. The results showed that the mean age of the participants was 15.83 ± 1.93 years and the mean household crowding index of the participants was 1.39 ± 1.59. The mean scales scores were as follows: LWDS (3.64 ± 6.54), FTND (1.39 ± 2.45), AUDIT (1.83 ± 5.07) and EAT-26 (16.79 ± 12.36). When dividing the EAT score into two categories according to the median (=13), the results showed that 185 (47.6%) of the adolescents had inappropriate eating attitudes (scores ≥14).

### Bivariate analysis

Higher age and higher BMI were significantly associated with higher problematic alcohol use. Higher EAT-26 scores (more inappropriate eating) were significantly associated with higher waterpipe and cigarette dependence and problematic alcohol use (Table 1).

**Table 1:** Bivariate analysis of factors associated with the waterpipe and cigarette dependence and problematic alcohol use

Variable	LWDS-11 score	FTND score	AUDIT score
Age	0.092	−0.086	0.159^b^
Body Mass Index	0.012	0.058	0.104^c^
House crowding index	0.078	0.047	−0.028
EAT-26 score	0.180^a^	0.118^c^	0.138^b^

*LWDS* Lebanese Waterpipe Dependence Scale, *FTND* Fagerstrom Nicotine Dependence Scale, *AUDIT* Alcohol use disorder identification test, *EAT* Eating attitude test; ^a^ *p* < 0.001; ^b^ *p* < 0.01; ^c^ *p* < 0.05; numbers correspond to the correlations coefficients (rho) obtained from the Spearman correlation test.

### Adjusted means

**Fig. 1:**
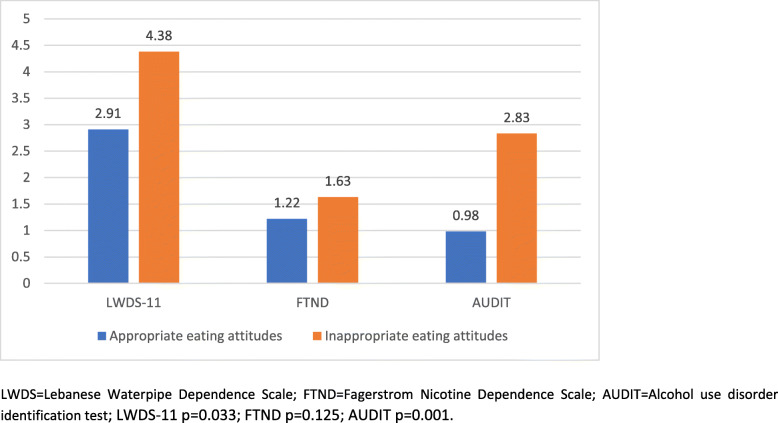
Mean values of the LWDS-11, FTND and AUDIT scores according to the presence/absence of inappropriate vs appropriate eating attitudes among adolescents, after adjusting for the covariates. LWDS = Lebanese Waterpipe Dependence Scale; FTND=Fagerstrom Nicotine Dependence Scale; AUDIT = Alcohol use disorder identification test; LWDS-11 *p* = 0.033; FTND *p* = 0.125; AUDIT *p* = 0.001

The means of the LWDS-11, FTND and AUDIT scores, according to having inappropriate vs appropriate eating attitudes, after adjusting for the covariates (age, household crowding index and BMI), are shown in Fig. 1. The results showed that inappropriate eating attitudes were significantly associated with higher LWDS-11 scores (more waterpipe dependence) and higher AUDIT scores (more problematic alcohol use) in adolescents, but not cigarette dependence.

### Multivariate analysis

The MANCOVA analysis was performed taking the addiction scales as the dependent variables and the EAT-26 score as an independent variable, adjusting for the covariates (age, BMI and household crowding index). Higher EAT-26 scores (more inappropriate eating attitudes) were significantly associated with higher waterpipe dependence (B = 0.11) and more problematic alcohol use (B = 0.08) but not cigarette dependence (Table 2).

**Table 2:** Multivariable analysis (MANCOVA)

Model 1: LWDS score taken as the dependent variable			
Variable	Beta	***p***	95% CI
Age	0.24	0.238	−0.16-0.65
House crowding index	0.31	0.523	−0.64-1.26
BMI	0.02	0.780	−0.12-0.16
EAT-26 score	0.11	**< 0.001**	0.06–0.17
**Model 2: FTND score taken as the dependent variable.**			
Age	0.05	0.561	−0.11-0.21
House crowding index	0.14	0.462	−0.23-0.51
BMI	0.04	0.196	−0.02-0.09
EAT-26 score	0.02	0.102	−0.004-0.04
**Model 3: AUDIT score taken as the dependent variable.**			
Age	0.14	0.392	−0.18-0.46
House crowding index	−0.26	0.503	−1.01-0.50
BMI	0.10	0.083	−0.01-0.21
EAT-26 score	0.08	**< 0.001**	0.04–0.12

*LWDS* Lebanese Waterpipe Dependence Scale, *FTND* Fagerstrom Nicotine Dependence Scale, *AUDIT* Alcohol use disorder identification test, *EAT* Eating attitude test, *BMI* Body Mass Index, numbers in bold indicate significant *p*-values.

## Discussion

This study aimed to investigate the relationship between inappropriate eating habits and one’s dependency on smoking – cigarettes and waterpipe – and drinking alcohol. It focused on a sample of males since few studies have investigated the effect of disordered eating habits in males. Furthermore, it focused on male adolescents since this age group is susceptible to drinking and smoking in Lebanon due to the lack of law implementation in addition to the misconception adolescents have in regards to perceiving different weight statuses [50]. Our results found that 47.6% of the interviewed male adolescents experience inappropriate eating behaviors. A recent study found that the prevalence of disordered eating habits among females is 50.7% [3]. The similarity in percentages highlights the importance of exploring the effect of this issue on different areas of male adolescents’ lives as well.

An increase in inappropriate eating habits was found to be correlated with waterpipe dependence in Lebanon; however, it had no relation with cigarette smoking. A study in Jordan also found that waterpipe-only smokers and dual smokers have unhealthy eating habits in comparison to cigarette-only smokers [51]. While multiple studies carried out show an association between inappropriate eating habits and an increase in smoking tobacco [52–54], a study in Jordan found that the prevalence of adolescents smoking only the waterpipe was 21.1% while those smoking only cigarettes was 6.7% [55].

The results we established regarding Lebanese male adolescents with inappropriate eating habit showing a waterpipe dependence, but not a cigarette dependence might be due to the increased popularity of waterpipes. It is important to mention that waterpipes are perceived as more socially acceptable than cigarettes [56, 57]. Waterpipe smoking among adolescents is a rising phenomenon in Lebanon and worldwide [28, 55]. Hence, this should be further investigated especially since the engagement of adolescents in disordered eating at their age puts them at risk of developing eating disorder or other eating behaviors that are unhealthy.

Studies also suggest that mostly, people tend to consider waterpipe to be less harmful and/or addictive than cigarettes [56]. However, one head of an unflavored waterpipe has actually been found to have the amount of nicotine found in 70 cigarettes [57]. In addition, nicotine has been found to have a suppressing effect on one’s appetite [58]. That could explain the relationship between waterpipe smoking and the manifestation of disordered eating habits, as adolescents strive to these measures in hope of losing weight. Not only has smoking tobacco been associated with increased physical inactivity [59], but also waterpipe smoking has been found to be associated with an increased risk of developing a high BMI and obesity in adults in comparison to non-smoking [60]. People seem to believe that these factors outweigh the consequences smoking - paired with disordered eating - has on one’s health, such as increasing risk of esophageal cancer, lung cancer or other cardiovascular diseases [61, 62].

As for alcohol drinking, our studies suggest that more disordered eating behaviors were associated with the consumption of higher amounts of alcohol. Most studies have found similar results where inappropriate eating habits lead to negative health habits such as drinking and smoking, as aforementioned [52–54]. However, a study suggested that males tend to want to be muscular and lean rather than lose weight [63], as opposed to what females tend to achieve [64]. Another study found that males who wanted to gain weight were more likely to engage in binge drinking and alcohol use than the males who wanted to lose weight [65]. The relationship between alcohol use and disordered eating should be further investigated, as there could be several reasons behind the link. In addition, the link could be due to peer pressure and adolescents wanting to social conform within their groups by drinking, paired with having a well-perceived body shape [54]. The term ‘drunkorexia’ has even been coined for the practice of dieting when drinking alcohol was planned, as means of decreasing the number of calories consumed within that day to prevent weight gain [66]. Moreover, some studies found evidence where disordered eating and substance abuse – including alcohol and tobacco – had overlapping genetic underpinnings [67]. In addition, the reason underlying the association between disordered eating and alcohol and/or tobacco consumption could be one’s way of coping with stress [68], as both are avoidance mechanisms [21] and adolescents would reduce their negative feelings through eating and/or drinking [69].

## Implications to practice

Our findings point out the importance of developing national awareness campaigns in different regions of the country, as male adolescents -like female adolescents- need attention on the matter of disordered eating and substance consumption. The foundations of the projects should start from home and expand to schools. Thus, there is a need to stress on having health promoting schools (HPS), where all stakeholders show responsibility in health matters of school-aged children in general and male adolescents in particular. HPS would cover a multitude of topics, including cigarette smoking and waterpipe and alcohol consumption among other topics. The schools may prioritize these areas over others, without neglecting the importance of each of HPS components. In other words, harm reduction is a key player in developing healthy individuals, starting with adolescents, as the behavior of the teenagers will definitely shape their future including their health.

## Limitations and strengths

This study has some limitations; it faces is the focus on disordered eating behaviors in general instead of focusing on specific ones. Moreover, the association between the disordered eating patterns and drinking or smoking could have several underlying factors, such as depression or anxiety, so psychological factors should have been investigated. The scales used to assess smoking dependence are not validated in Lebanon. The sample enrolled in this study is small in size. A selection bias is possible because of the refusal rate. In addition, a sample of only 5 schools poses a limitation. A residual confounding bias is also possible since not all factors associated with alcohol and smoking dependence were taken into consideration in this study; of these factors are low earnings, weaker labor market attachments, and genetic factors. Finally, the data were self-reported as questionnaires were handed out to the participants. Hence, our study might exhibit some information bias due to a participant’s misinterpretation of a question decreasing the validity of his response. The results of this study cannot be generalized to the whole population; further larger studies taking all these limitations and tackling both genders are needed.

This study offers an added value in terms of the sample interviewed since most studies related to the effects of disordered eating on one’s life are focused on women. In addition to our sample being exclusively male, it focused on adolescent males since experiencing disordered eating at this age group increases risk of developing an eating disorder at later stages in life. This age group is also susceptible to misconceptions regarding weight status. Furthermore, our study focuses on the effects disordered eating has on unhealthy behaviors such as smoking and drinking. Hence, appropriate measures to prevent disordered eating and the incidence of smoking and drinking should be put into place.

## Conclusion

This study revealed an association between disordered eating among male adolescents and increased drinking and smoking waterpipe, but not cigarette smoking. In Lebanon, the engagement of adolescents in alcohol drinking and waterpipe smoking is frequent as they are easily accessible due to the lack of law enforcement in the country. Hence, these results are of particular importance in Lebanon as this might tip the scale in favor of the perceived benefits smoking and alcohol drinking have – such as reducing stress and decreasing appetite – where people with disordered eating habits would disregard the long-term consequences of these eating behaviors paired with smoking and alcohol. Furthermore, further investigation should be done on the role of one’s psychology as a factor underlying the relationship between the exhibition of disordered eating and smoking and alcohol drinking in Lebanon, as means to prevent the manifestation of these habits.

## Data Availability

All data generated or analyzed during this study are not publicly available to maintain the privacy of the individuals’ identities. The dataset supporting the conclusions is available upon request to the corresponding author.
